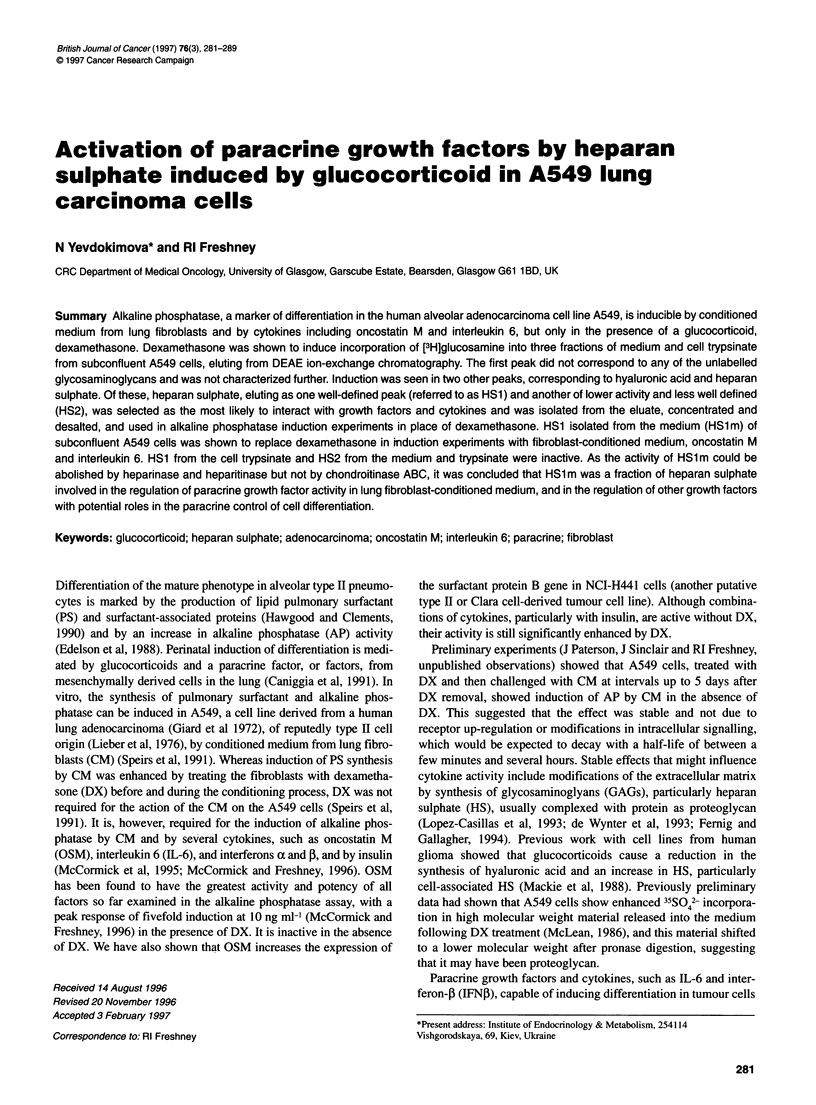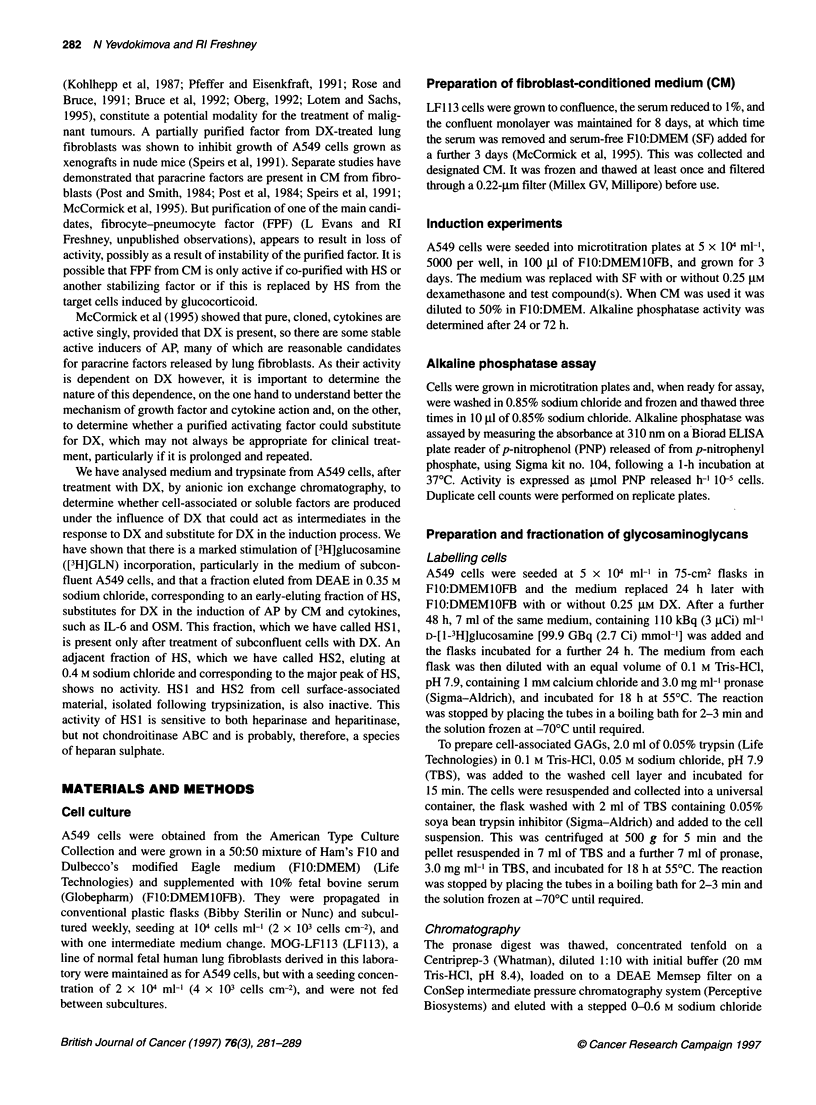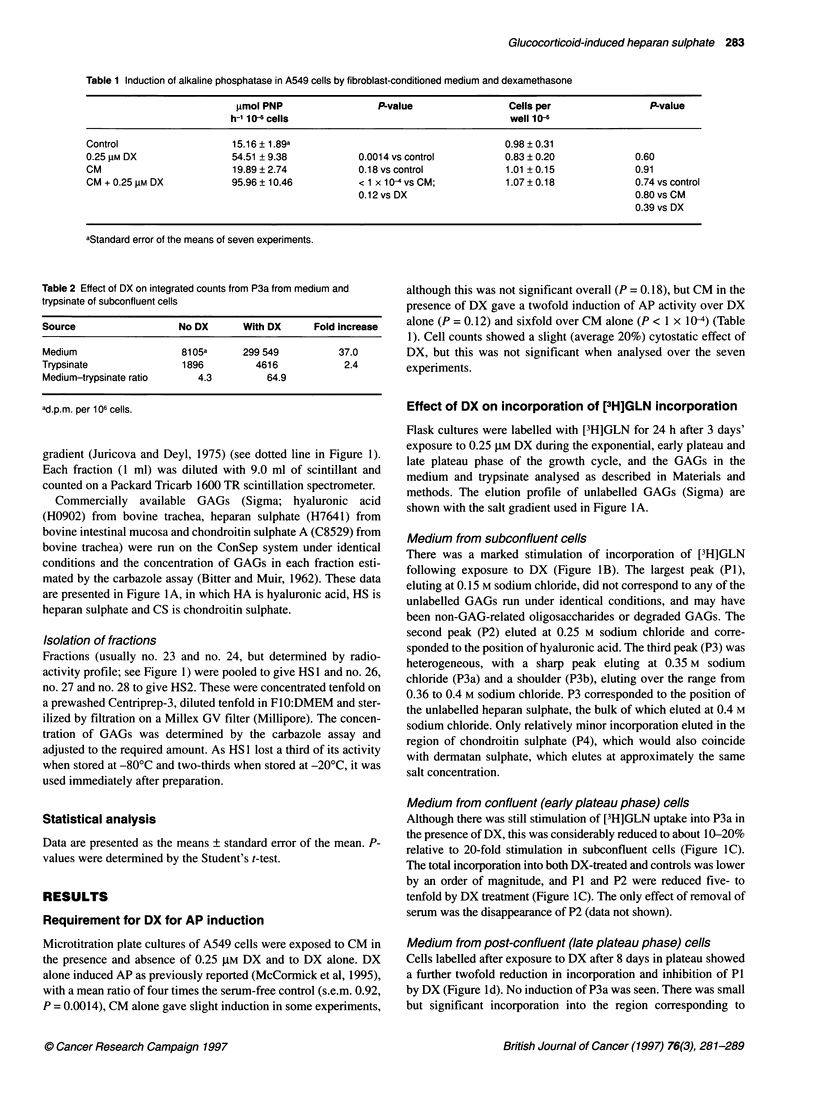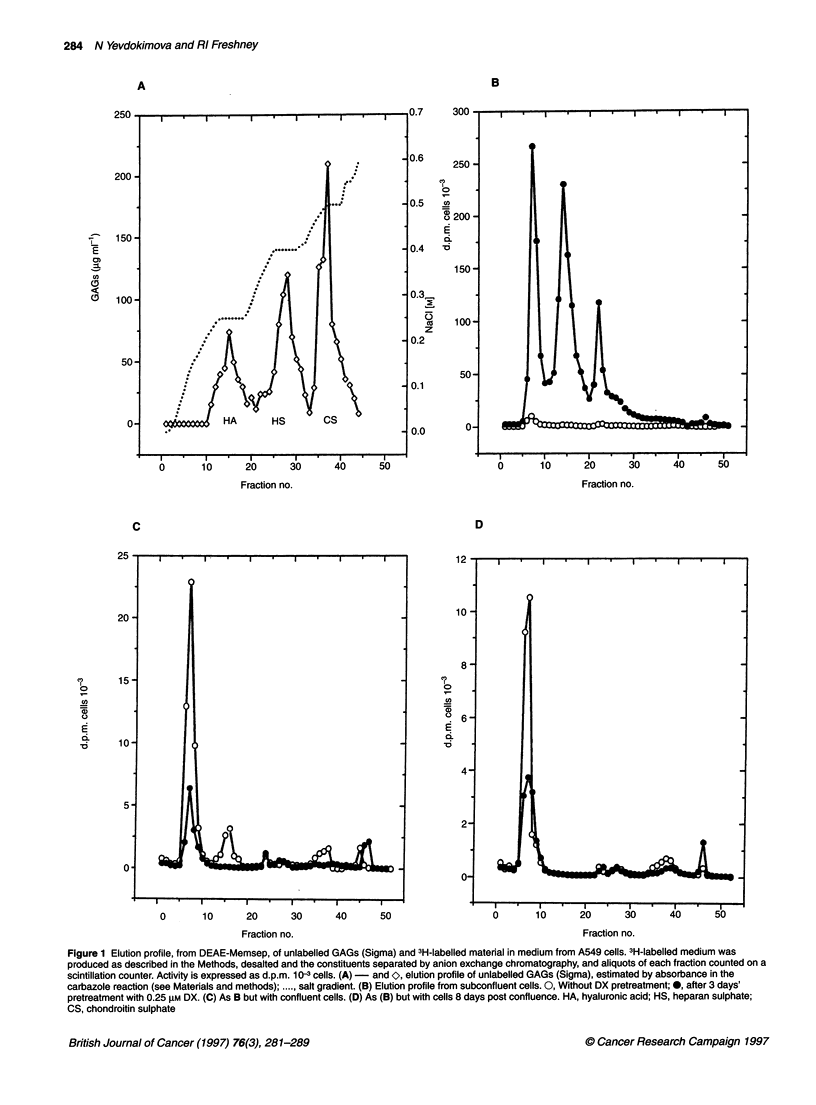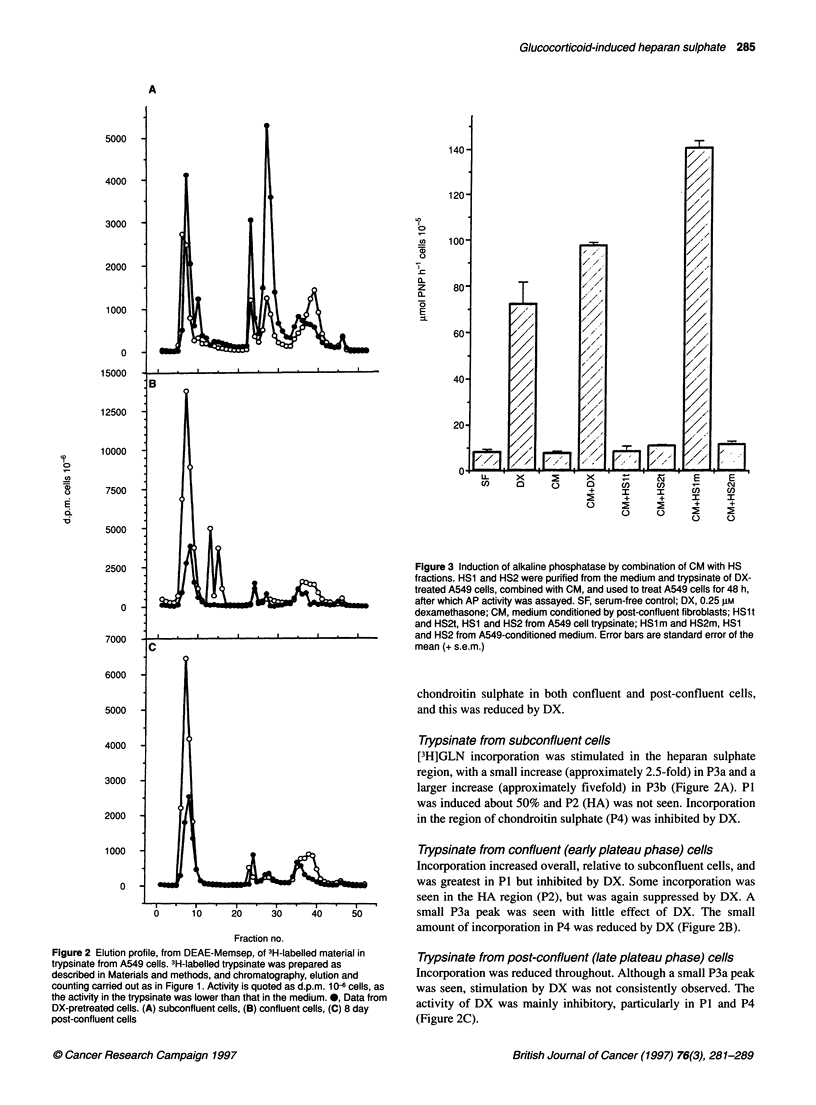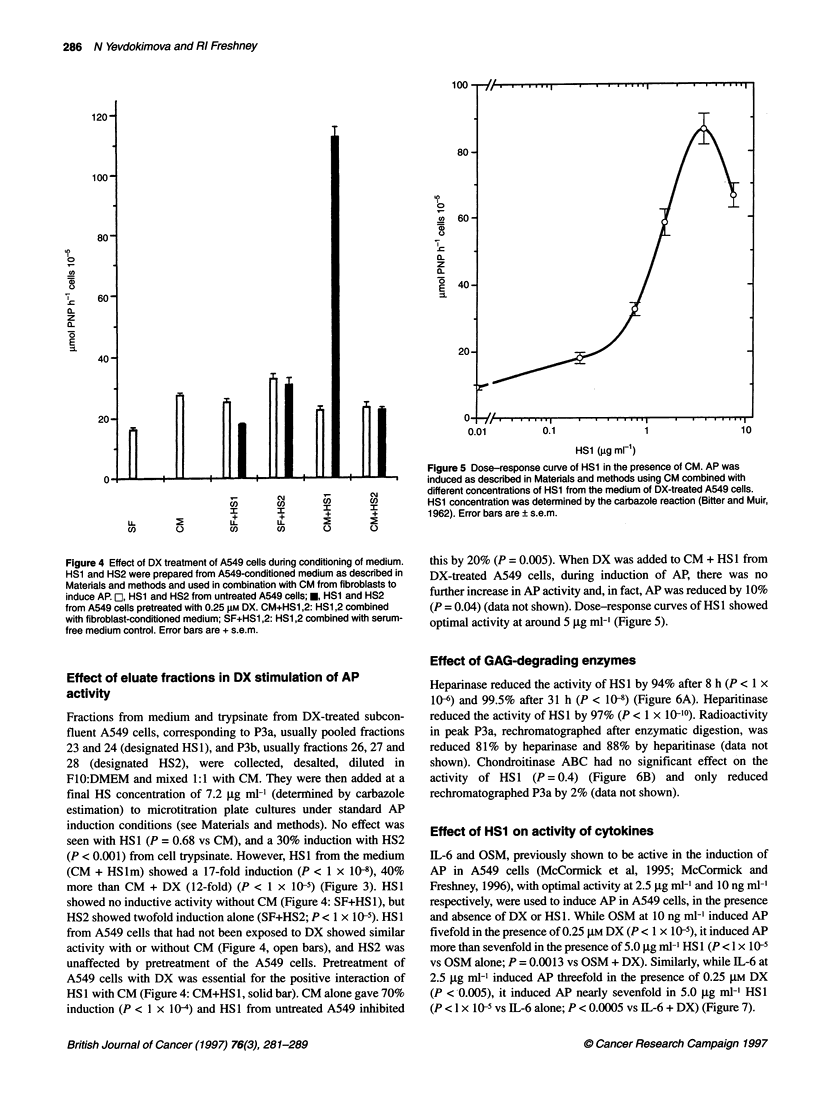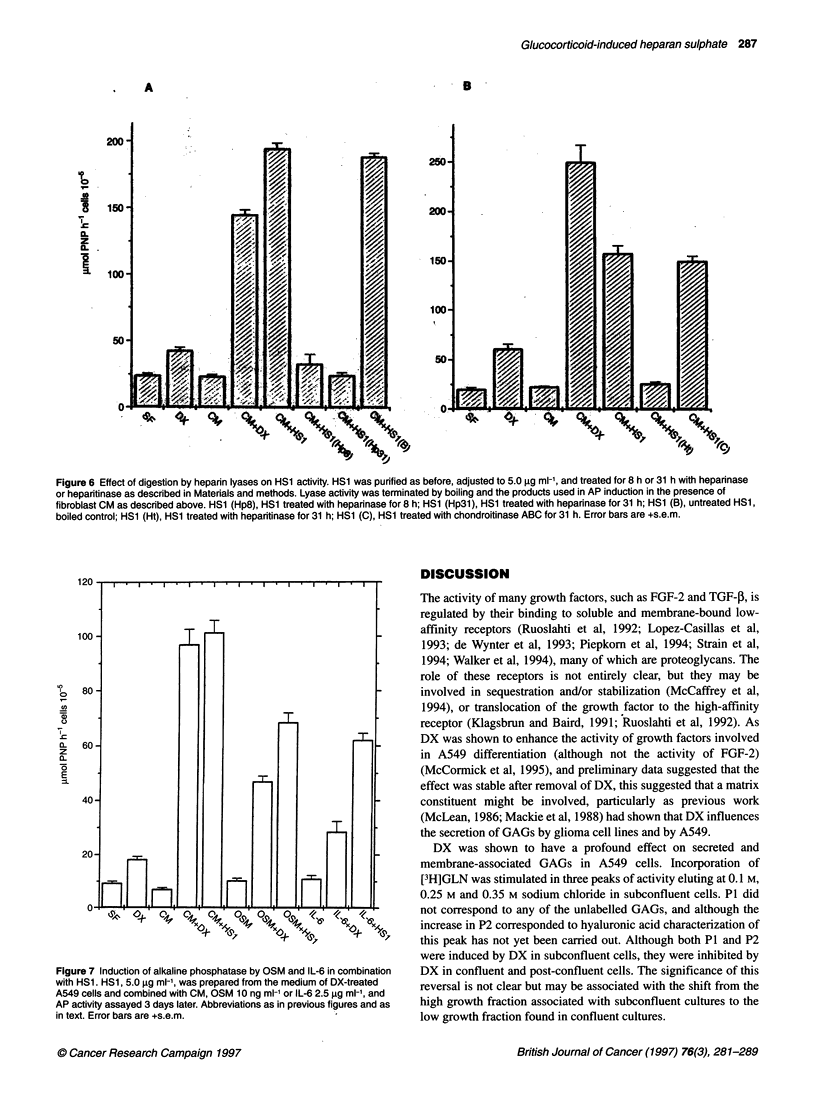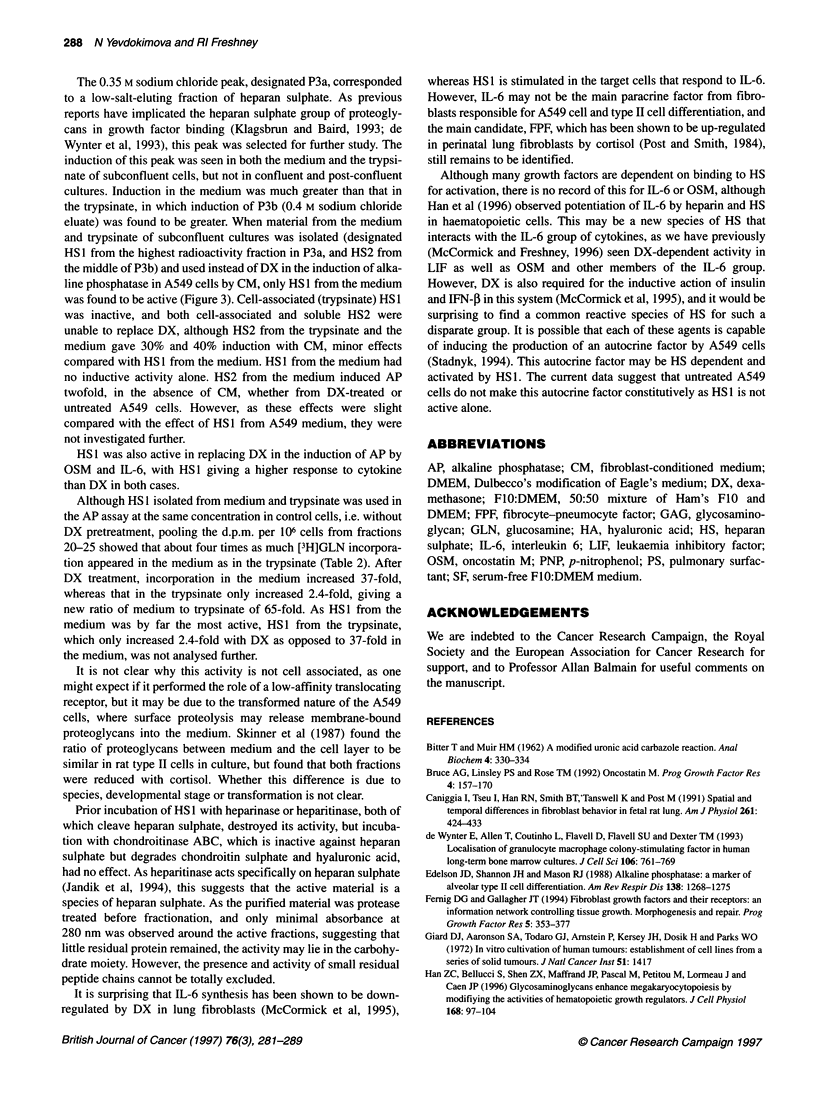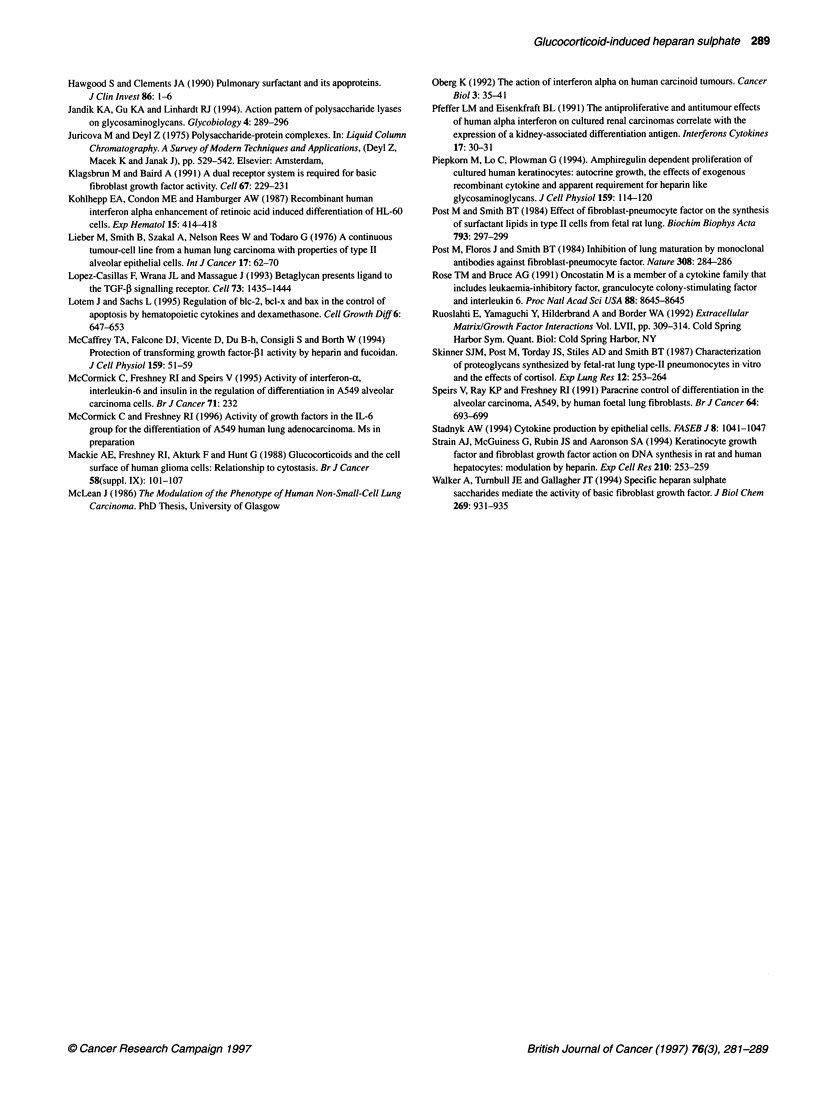# Activation of paracrine growth factors by heparan sulphate induced by glucocorticoid in A549 lung carcinoma cells.

**DOI:** 10.1038/bjc.1997.380

**Published:** 1997

**Authors:** N. Yevdokimova, R. I. Freshney

**Affiliations:** CRC Department of Medical Oncology, University of Glasgow, Bearsden, UK.

## Abstract

Alkaline phosphatase, a marker of differentiation in the human alveolar adenocarcinoma cell line A549, is inducible by conditioned medium from lung fibroblasts and by cytokines including oncostatin M and interleukin 6, but only in the presence of a glucocorticoid, dexamethasone. Dexamethasone was shown to induce incorporation of [3H]glucosamine into three fractions of medium and cell trypsinate from subconfluent A549 cells, eluting from DEAE ion-exchange chromatography. The first peak did not correspond to any of the unlabelled glycosaminoglycans and was not characterized further. Induction was seen in two other peaks, corresponding to hyaluronic acid and heparan sulphate. Of these, heparan sulphate, eluting as one well-defined peak (referred to as HS1) and another of lower activity and less well defined (HS2), was selected as the most likely to interact with growth factors and cytokines and was isolated from the eluate, concentrated and desalted, and used in alkaline phosphatase induction experiments in place of dexamethasone. HS1 isolated from the medium (HS1m) of subconfluent A549 cells was shown to replace dexamethasone in induction experiments with fibroblast-conditioned medium, oncostatin M and interleukin 6. HS1 from the cell trypsinate and HS2 from the medium and trypsinate were inactive. As the activity of HS1m could be abolished by heparinase and heparitinase but not by chondroitinase ABC, it was concluded that HS1m was a fraction of heparan sulphate involved in the regulation of paracrine growth factor activity in lung fibroblast-conditioned medium, and in the regulation of other growth factors with potential roles in the paracrine control of cell differentiation.


British Journal of Cancer (1997) 76(3), 281–289
© 1997 Cancer Research Campaign

### Activation of paracrine growth factors by heparan sulphate induced by glucocorticoid in A549 lung carcinoma cells

**N Yevdokimova* and RI Freshney**

CRC Department of Medical Oncology, University of Glasgow, Garscube Estate, Bearsden, Glasgow G61 1BD, UK

**Summary** Alkaline phosphatase, a marker of differentiation in the human alveolar adenocarcinoma cell line A549, is inducible by conditioned medium from lung fibroblasts and by cytokines including oncostatin M and interleukin 6, but only in the presence of a glucocorticoid, dexamethasone. Dexamethasone was shown to induce incorporation of [$^3$H]glucosamine into three fractions of medium and cell trypsinate from subconfluent A549 cells, eluting from DEAE ion-exchange chromatography. The first peak did not correspond to any of the unlabelled glycosaminoglycans and was not characterized further. Induction was seen in two other peaks, corresponding to hyaluronic acid and heparan sulphate. Of these, heparan sulphate, eluting as one well-defined peak (referred to as HS1) and another of lower activity and less well defined (HS2), was selected as the most likely to interact with growth factors and cytokines and was isolated from the eluate, concentrated and desalted, and used in alkaline phosphatase induction experiments in place of dexamethasone. HS1 isolated from the medium (HS1m) of subconfluent A549 cells was shown to replace dexamethasone in induction experiments with fibroblast-conditioned medium, oncostatin M and interleukin 6. HS1 from the cell trypsinate and HS2 from the medium and trypsinate were inactive. As the activity of HS1m could be abolished by heparinase and heparitinase but not by chondroitinase ABC, it was concluded that HS1m was a fraction of heparan sulphate involved in the regulation of paracrine growth factor activity in lung fibroblast-conditioned medium, and in the regulation of other growth factors with potential roles in the paracrine control of cell differentiation.

**Keywords:** glucocorticoid; heparan sulphate; adenocarcinoma; oncostatin M; interleukin 6; paracrine; fibroblast

Differentiation of the mature phenotype in alveolar type II pneumocytes is marked by the production of lipid pulmonary surfactant (PS) and surfactant-associated proteins (Hawgood and Clements, 1990) and by an increase in alkaline phosphatase (AP) activity (Edelson et al, 1988). Perinatal induction of differentiation is mediated by glucocorticoids and a paracrine factor, or factors, from mesenchymally derived cells in the lung (Caniggia et al, 1991). In vitro, the synthesis of pulmonary surfactant and alkaline phosphatase can be induced in A549, a cell line derived from a human lung adenocarcinoma (Giard et al 1972), of reputedly type II cell origin (Lieber et al, 1976), by conditioned medium from lung fibroblasts (CM) (Speirs et al, 1991). Whereas induction of PS synthesis by CM was enhanced by treating the fibroblasts with dexamethasone (DX) before and during the conditioning process, DX was not required for the action of the CM on the A549 cells (Speirs et al, 1991). It is, however, required for the induction of alkaline phosphatase by CM and by several cytokines, such as oncostatin M (OSM), interleukin 6 (IL-6), and interferons α and β, and by insulin (McCormick et al, 1995; McCormick and Freshney, 1996). OSM has been found to have the greatest activity and potency of all factors so far examined in the alkaline phosphatase assay, with a peak response of fivefold induction at 10 ng ml$^{-1}$ (McCormick and Freshney, 1996) in the presence of DX. It is inactive in the absence of DX. We have also shown that OSM increases the expression of the surfactant protein B gene in NCI-H441 cells (another putative type II or Clara cell-derived tumour cell line). Although combinations of cytokines, particularly with insulin, are active without DX, their activity is still significantly enhanced by DX.

Preliminary experiments (J Paterson, J Sinclair and RI Freshney, unpublished observations) showed that A549 cells, treated with DX and then challenged with CM at intervals up to 5 days after DX removal, showed induction of AP by CM in the absence of DX. This suggested that the effect was stable and not due to receptor up-regulation or modifications in intracellular signalling, which would be expected to decay with a half-life of between a few minutes and several hours. Stable effects that might influence cytokine activity include modifications of the extracellular matrix by synthesis of glycosaminoglyans (GAGs), particularly heparan sulphate (HS), usually complexed with protein as proteoglycan (Lopez-Casillas et al, 1993; de Wynter et al, 1993; Fernig and Gallagher, 1994). Previous work with cell lines from human glioma showed that glucocorticoids cause a reduction in the synthesis of hyaluronic acid and an increase in HS, particularly cell-associated HS (Mackie et al, 1988). Previously preliminary data had shown that A549 cells show enhanced $^{35}SO_4^{2-}$ incorporation in high molecular weight material released into the medium following DX treatment (McLean, 1986), and this material shifted to a lower molecular weight after pronase digestion, suggesting that it may have been proteoglycan.

Paracrine growth factors and cytokines, such as IL-6 and interferon-β (IFNβ), capable of inducing differentiation in tumour cells

*Received 14 August 1996*
*Revised 20 November 1996*
*Accepted 3 February 1997*

*Correspondence to:* RI Freshney

*Present address: Institute of Endocrinology & Metabolism, 254114 Vishgorodskaya, 69, Kiev, Ukraine

(Kohlhepp et al, 1987; Pfeffer and Eisenkfraft, 1991; Rose and Bruce, 1991; Bruce et al, 1992; Oberg, 1992; Lotem and Sachs, 1995), constitute a potential modality for the treatment of malignant tumours. A partially purified factor from DX-treated lung fibroblasts was shown to inhibit growth of A549 cells grown as xenografts in nude mice (Speirs et al, 1991). Separate studies have demonstrated that paracrine factors are present in CM from fibroblasts (Post and Smith, 1984; Post et al, 1984; Speirs et al, 1991; McCormick et al, 1995). But purification of one of the main candidates, fibrocyte–pneumocyte factor (FPF) (L Evans and RI Freshney, unpublished observations), appears to result in loss of activity, possibly as a result of instability of the purified factor. It is possible that FPF from CM is only active if co-purified with HS or another stabilizing factor or if this is replaced by HS from the target cells induced by glucocorticoid.

McCormick et al (1995) showed that pure, cloned, cytokines are active singly, provided that DX is present, so there are some stable active inducers of AP, many of which are reasonable candidates for paracrine factors released by lung fibroblasts. As their activity is dependent on DX however, it is important to determine the nature of this dependence, on the one hand to understand better the mechanism of growth factor and cytokine action and, on the other, to determine whether a purified activating factor could substitute for DX, which may not always be appropriate for clinical treatment, particularly if it is prolonged and repeated.

We have analysed medium and trypsinate from A549 cells, after treatment with DX, by anionic ion exchange chromatography, to determine whether cell-associated or soluble factors are produced under the influence of DX that could act as intermediates in the response to DX and substitute for DX in the induction process. We have shown that there is a marked stimulation of [$^3$H]glucosamine ([$^3$H]GLN) incorporation, particularly in the medium of subconfluent A549 cells, and that a fraction eluted from DEAE in 0.35 M sodium chloride, corresponding to an early-eluting fraction of HS, substitutes for DX in the induction of AP by CM and cytokines, such as IL-6 and OSM. This fraction, which we have called HS1, is present only after treatment of subconfluent cells with DX. An adjacent fraction of HS, which we have called HS2, eluting at 0.4 M sodium chloride and corresponding to the major peak of HS, shows no activity. HS1 and HS2 from cell surface-associated material, isolated following trypsinization, is also inactive. This activity of HS1 is sensitive to both heparinase and heparitinase, but not chondroitinase ABC and is probably, therefore, a species of heparan sulphate.

### MATERIALS AND METHODS

### Cell culture

A549 cells were obtained from the American Type Culture Collection and were grown in a 50:50 mixture of Ham's F10 and Dulbecco's modified Eagle medium (F10:DMEM) (Life Technologies) and supplemented with 10% fetal bovine serum (Globepharm) (F10:DMEM10FB). They were propagated in conventional plastic flasks (Bibby Sterilin or Nunc) and subcultured weekly, seeding at $10^4$ cells ml$^{-1}$ ($2 \times 10^3$ cells cm$^{-2}$), and with one intermediate medium change. MOG-LF113 (LF113), a line of normal fetal human lung fibroblasts derived in this laboratory were maintained as for A549 cells, but with a seeding concentration of $2 \times 10^4$ ml$^{-1}$ ($4 \times 10^3$ cells cm$^{-2}$), and were not fed between subcultures.

### Preparation of fibroblast-conditioned medium (CM)

LF113 cells were grown to confluence, the serum reduced to 1%, and the confluent monolayer was maintained for 8 days, at which time the serum was removed and serum-free F10:DMEM (SF) added for a further 3 days (McCormick et al, 1995). This was collected and designated CM. It was frozen and thawed at least once and filtered through a 0.22-μm filter (Millex GV, Millipore) before use.

### Induction experiments

A549 cells were seeded into microtitration plates at $5 \times 10^4$ ml$^{-1}$, 5000 per well, in 100 μl of F10:DMEM10FB, and grown for 3 days. The medium was replaced with SF with or without 0.25 μM dexamethasone and test compound(s). When CM was used it was diluted to 50% in F10:DMEM. Alkaline phosphatase activity was determined after 24 or 72 h.

### Alkaline phosphatase assay

Cells were grown in microtitration plates and, when ready for assay, were washed in 0.85% sodium chloride and frozen and thawed three times in 10 μl of 0.85% sodium chloride. Alkaline phosphatase was assayed by measuring the absorbance at 310 nm on a Biorad ELISA plate reader of *p*-nitrophenol (PNP) released of from *p*-nitrophenyl phosphate, using Sigma kit no. 104, following a 1-h incubation at 37°C. Activity is expressed as μmol PNP released h$^{-1}$ $10^{-5}$ cells. Duplicate cell counts were performed on replicate plates.

### Preparation and fractionation of glycosaminoglycans

*Labelling cells*

A549 cells were seeded at $5 \times 10^4$ ml$^{-1}$ in 75-cm$^2$ flasks in F10:DMEM10FB and the medium replaced 24 h later with F10:DMEM10FB with or without 0.25 μM DX. After a further 48 h, 7 ml of the same medium, containing 110 kBq (3 μCi) ml$^{-1}$ D-[1-$^3$H]glucosamine [99.9 GBq (2.7 Ci) mmol$^{-1}$] was added and the flasks incubated for a further 24 h. The medium from each flask was then diluted with an equal volume of 0.1 M Tris-HCl, pH 7.9, containing 1 mM calcium chloride and 3.0 mg ml$^{-1}$ pronase (Sigma–Aldrich), and incubated for 18 h at 55°C. The reaction was stopped by placing the tubes in a boiling bath for 2–3 min and the solution frozen at –70°C until required.

To prepare cell-associated GAGs, 2.0 ml of 0.05% trypsin (Life Technologies) in 0.1 M Tris-HCl, 0.05 M sodium chloride, pH 7.9 (TBS), was added to the washed cell layer and incubated for 15 min. The cells were resuspended and collected into a universal container, the flask washed with 2 ml of TBS containing 0.05% soya bean trypsin inhibitor (Sigma–Aldrich) and added to the cell suspension. This was centrifuged at 500 *g* for 5 min and the pellet resuspended in 7 ml of TBS and a further 7 ml of pronase, 3.0 mg ml$^{-1}$ in TBS, and incubated for 18 h at 55°C. The reaction was stopped by placing the tubes in a boiling bath for 2–3 min and the solution frozen at –70°C until required.

*Chromatography*

The pronase digest was thawed, concentrated tenfold on a Centriprep-3 (Whatman), diluted 1:10 with initial buffer (20 mM Tris-HCl, pH 8.4), loaded on to a DEAE Memsep filter on a ConSep intermediate pressure chromatography system (Perceptive Biosystems) and eluted with a stepped 0–0.6 M sodium chloride

 © Cancer Research Campaign 1997

**Table 1** Induction of alkaline phosphatase in A549 cells by fibroblast-conditioned medium and dexamethasone

| | μmol PNP $h^{-1}$ $10^{-5}$ cells | P-value | Cells per well $10^{-5}$ | P-value |
|---|---|---|---|---|
| Control | 15.16 ± 1.89[a] | | 0.98 ± 0.31 | |
| 0.25 μM DX | 54.51 ± 9.38 | 0.0014 vs control | 0.83 ± 0.20 | 0.60 |
| CM | 19.89 ± 2.74 | 0.18 vs control | 1.01 ± 0.15 | 0.91 |
| CM + 0.25 μM DX | 95.96 ± 10.46 | < 1 × $10^{-4}$ vs CM; 0.12 vs DX | 1.07 ± 0.18 | 0.74 vs control 0.80 vs CM 0.39 vs DX |

[a]Standard error of the means of seven experiments.

**Table 2** Effect of DX on integrated counts from P3a from medium and trypsinate of subconfluent cells

| Source | No DX | With DX | Fold increase |
|---|---|---|---|
| Medium | 8105[a] | 299 549 | 37.0 |
| Trypsinate | 1896 | 4616 | 2.4 |
| Medium–trypsinate ratio | 4.3 | 64.9 | |

[a]d.p.m. per $10^6$ cells.

gradient (Juricova and Deyl, 1975) (see dotted line in Figure 1). Each fraction (1 ml) was diluted with 9.0 ml of scintillant and counted on a Packard Tricarb 1600 TR scintillation spectrometer.

Commercially available GAGs (Sigma; hyaluronic acid (H0902) from bovine trachea, heparan sulphate (H7641) from bovine intestinal mucosa and chondroitin sulphate A (C8529) from bovine trachea) were run on the ConSep system under identical conditions and the concentration of GAGs in each fraction estimated by the carbazole assay (Bitter and Muir, 1962). These data are presented in Figure 1A, in which HA is hyaluronic acid, HS is heparan sulphate and CS is chondroitin sulphate.

### *Isolation of fractions*

Fractions (usually no. 23 and no. 24, but determined by radioactivity profile; see Figure 1) were pooled to give HS1 and no. 26, no. 27 and no. 28 to give HS2. These were concentrated tenfold on a prewashed Centriprep-3, diluted tenfold in F10:DMEM and sterilized by filtration on a Millex GV filter (Millipore). The concentration of GAGs was determined by the carbazole assay and adjusted to the required amount. As HS1 lost a third of its activity when stored at –80°C and two-thirds when stored at –20°C, it was used immediately after preparation.

### Statistical analysis

Data are presented as the means ± standard error of the mean. P-values were determined by the Student's *t*-test.

### RESULTS

### Requirement for DX for AP induction

Microtitration plate cultures of A549 cells were exposed to CM in the presence and absence of 0.25 μM DX and to DX alone. DX alone induced AP as previously reported (McCormick et al, 1995), with a mean ratio of four times the serum-free control (s.e.m. 0.92, $P = 0.0014$), CM alone gave slight induction in some experiments, although this was not significant overall ($P = 0.18$), but CM in the presence of DX gave a twofold induction of AP activity over DX alone ($P = 0.12$) and sixfold over CM alone ($P < 1 \times 10^{-4}$) (Table 1). Cell counts showed a slight (average 20%) cytostatic effect of DX, but this was not significant when analysed over the seven experiments.

### Effect of DX on incorporation of [$^3$H]GLN incorporation

Flask cultures were labelled with [$^3$H]GLN for 24 h after 3 days' exposure to 0.25 μM DX during the exponential, early plateau and late plateau phase of the growth cycle, and the GAGs in the medium and trypsinate analysed as described in Materials and methods. The elution profile of unlabelled GAGs (Sigma) are shown with the salt gradient used in Figure 1A.

### *Medium from subconfluent cells*

There was a marked stimulation of incorporation of [$^3$H]GLN following exposure to DX (Figure 1B). The largest peak (P1), eluting at 0.15 M sodium chloride, did not correspond to any of the unlabelled GAGs run under identical conditions, and may have been non-GAG-related oligosaccharides or degraded GAGs. The second peak (P2) eluted at 0.25 M sodium chloride and corresponded to the position of hyaluronic acid. The third peak (P3) was heterogeneous, with a sharp peak eluting at 0.35 M sodium chloride (P3a) and a shoulder (P3b), eluting over the range from 0.36 to 0.4 M sodium chloride. P3 corresponded to the position of the unlabelled heparan sulphate, the bulk of which eluted at 0.4 M sodium chloride. Only relatively minor incorporation eluted in the region of chondroitin sulphate (P4), which would also coincide with dermatan sulphate, which elutes at approximately the same salt concentration.

### *Medium from confluent (early plateau phase) cells*

Although there was still stimulation of [$^3$H]GLN uptake into P3a in the presence of DX, this was considerably reduced to about 10–20% relative to 20-fold stimulation in subconfluent cells (Figure 1C). The total incorporation into both DX-treated and controls was lower by an order of magnitude, and P1 and P2 were reduced five- to tenfold by DX treatment (Figure 1C). The only effect of removal of serum was the disappearance of P2 (data not shown).

### *Medium from post-confluent (late plateau phase) cells*

Cells labelled after exposure to DX after 8 days in plateau showed a further twofold reduction in incorporation and inhibition of P1 by DX (Figure 1d). No induction of P3a was seen. There was small but significant incorporation into the region corresponding to

© Cancer Research Campaign 1997

Figure 1 Elution profile, from DEAE-Memsep, of unlabelled GAGs (Sigma) and $^3$H-labelled material in medium from A549 cells. $^3$H-labelled medium was produced as described in the Methods, desalted and the constituents separated by anion exchange chromatography, and aliquots of each fraction counted on a scintillation counter. Activity is expressed as d.p.m. $10^{-3}$ cells. (**A**) — and ◇, elution profile of unlabelled GAGs (Sigma), estimated by absorbance in the carbazole reaction (see Materials and methods); ....., salt gradient. (**B**) Elution profile from subconfluent cells. ○, Without DX pretreatment; ●, after 3 days' pretreatment with 0.25 μM DX. (**C**) As **B** but with confluent cells. (**D**) As (**B**) but with cells 8 days post confluence. HA, hyaluronic acid; HS, heparan sulphate; CS, chondroitin sulphate

© Cancer Research Campaign 1997

**Figure 2** Elution profile, from DEAE-Memsep, of $^3$H-labelled material in trypsinate from A549 cells. $^3$H-labelled trypsinate was prepared as described in Materials and methods, and chromatography, elution and counting carried out as in Figure 1. Activity is quoted as d.p.m. $10^{-6}$ cells, as the activity in the trypsinate was lower than that in the medium. ●, Data from DX-pretreated cells. (**A**) subconfluent cells, (**B**) confluent cells, (**C**) 8 day post-confluent cells

**Figure 3** Induction of alkaline phosphatase by combination of CM with HS fractions. HS1 and HS2 were purified from the medium and trypsinate of DX-treated A549 cells, combined with CM, and used to treat A549 cells for 48 h, after which AP activity was assayed. SF, serum-free control; DX, 0.25 μM dexamethasone; CM, medium conditioned by post-confluent fibroblasts; HS1t and HS2t, HS1 and HS2 from A549 cell trypsinate; HS1m and HS2m, HS1 and HS2 from A549-conditioned medium. Error bars are standard error of the mean (+ s.e.m.)

chondroitin sulphate in both confluent and post-confluent cells, and this was reduced by DX.

### *Trypsinate from subconfluent cells*

[$^3$H]GLN incorporation was stimulated in the heparan sulphate region, with a small increase (approximately 2.5-fold) in P3a and a larger increase (approximately fivefold) in P3b (Figure 2A). P1 was induced about 50% and P2 (HA) was not seen. Incorporation in the region of chondroitin sulphate (P4) was inhibited by DX.

### *Trypsinate from confluent (early plateau phase) cells*

Incorporation increased overall, relative to subconfluent cells, and was greatest in P1 but inhibited by DX. Some incorporation was seen in the HA region (P2), but was again suppressed by DX. A small P3a peak was seen with little effect of DX. The small amount of incorporation in P4 was reduced by DX (Figure 2B).

### *Trypsinate from post-confluent (late plateau phase) cells*

Incorporation was reduced throughout. Although a small P3a peak was seen, stimulation by DX was not consistently observed. The activity of DX was mainly inhibitory, particularly in P1 and P4 (Figure 2C).

© Cancer Research Campaign 1997

**Figure 4** Effect of DX treatment of A549 cells during conditioning of medium. HS1 and HS2 were prepared from A549-conditioned medium as described in Materials and methods and used in combination with CM from fibroblasts to induce AP. □, HS1 and HS2 from untreated A549 cells; ■, HS1 and HS2 from A549 cells pretreated with 0.25 μM DX. CM+HS1,2: HS1,2 combined with fibroblast-conditioned medium; SF+HS1,2: HS1,2 combined with serum-free medium control. Error bars are + s.e.m.

**Figure 5** Dose–response curve of HS1 in the presence of CM. AP was induced as described in Materials and methods using CM combined with different concentrations of HS1 from the medium of DX-treated A549 cells. HS1 concentration was determined by the carbazole reaction (Bitter and Muir, 1962). Error bars are ± s.e.m.

### Effect of eluate fractions in DX stimulation of AP activity

Fractions from medium and trypsinate from DX-treated subconfluent A549 cells, corresponding to P3a, usually pooled fractions 23 and 24 (designated HS1), and P3b, usually fractions 26, 27 and 28 (designated HS2), were collected, desalted, diluted in F10:DMEM and mixed 1:1 with CM. They were then added at a final HS concentration of 7.2 μg $ml^{-1}$ (determined by carbazole estimation) to microtitration plate cultures under standard AP induction conditions (see Materials and methods). No effect was seen with HS1 ($P = 0.68$ vs CM), and a 30% induction with HS2 ($P < 0.001$) from cell trypsinate. However, HS1 from the medium (CM + HS1m) showed a 17-fold induction ($P < 1 \times 10^{-8}$), 40% more than CM + DX (12-fold) ($P < 1 \times 10^{-5}$) (Figure 3). HS1 showed no inductive activity without CM (Figure 4: SF+HS1), but HS2 showed twofold induction alone (SF+HS2; $P < 1 \times 10^{-5}$). HS1 from A549 cells that had not been exposed to DX showed similar activity with or without CM (Figure 4, open bars), and HS2 was unaffected by pretreatment of the A549 cells. Pretreatment of A549 cells with DX was essential for the positive interaction of HS1 with CM (Figure 4: CM+HS1, solid bar). CM alone gave 70% induction ($P < 1 \times 10^{-4}$) and HS1 from untreated A549 inhibited this by 20% ($P = 0.005$). When DX was added to CM + HS1 from DX-treated A549 cells, during induction of AP, there was no further increase in AP activity and, in fact, AP was reduced by 10% ($P = 0.04$) (data not shown). Dose–response curves of HS1 showed optimal activity at around 5 μg $ml^{-1}$ (Figure 5).

### Effect of GAG-degrading enzymes

Heparinase reduced the activity of HS1 by 94% after 8 h ($P < 1 \times 10^{-6}$) and 99.5% after 31 h ($P < 10^{-8}$) (Figure 6A). Heparitinase reduced the activity of HS1 by 97% ($P < 1 \times 10^{-10}$). Radioactivity in peak P3a, rechromatographed after enzymatic digestion, was reduced 81% by heparinase and 88% by heparitinase (data not shown). Chondroitinase ABC had no significant effect on the activity of HS1 ($P = 0.4$) (Figure 6B) and only reduced rechromatographed P3a by 2% (data not shown).

### Effect of HS1 on activity of cytokines

IL-6 and OSM, previously shown to be active in the induction of AP in A549 cells (McCormick et al, 1995; McCormick and Freshney, 1996), with optimal activity at 2.5 μg $ml^{-1}$ and 10 ng $ml^{-1}$ respectively, were used to induce AP in A549 cells, in the presence and absence of DX or HS1. While OSM at 10 ng $ml^{-1}$ induced AP fivefold in the presence of 0.25 μM DX ($P < 1 \times 10^{-5}$), it induced AP more than sevenfold in the presence of 5.0 μg $ml^{-1}$ HS1 ($P < 1 \times 10^{-5}$ vs OSM alone; $P = 0.0013$ vs OSM + DX). Similarly, while IL-6 at 2.5 μg $ml^{-1}$ induced AP threefold in the presence of 0.25 μM DX ($P < 0.005$), it induced AP nearly sevenfold in 5.0 μg $ml^{-1}$ HS1 ($P < 1 \times 10^{-5}$ vs IL-6 alone; $P < 0.0005$ vs IL-6 + DX) (Figure 7).

© Cancer Research Campaign 1997

**Figure 6** Effect of digestion by heparin lyases on HS1 activity. HS1 was purified as before, adjusted to 5.0 μg ml$^{-1}$, and treated for 8 h or 31 h with heparinase or heparitinase as described in Materials and methods. Lyase activity was terminated by boiling and the products used in AP induction in the presence of fibroblast CM as described above. HS1 (Hp8), HS1 treated with heparinase for 8 h; HS1 (Hp31), HS1 treated with heparinase for 31 h; HS1 (B), untreated HS1, boiled control; HS1 (Ht), HS1 treated with heparitinase for 31 h; HS1 (C), HS1 treated with chondroitinase ABC for 31 h. Error bars are +s.e.m.

**Figure 7** Induction of alkaline phosphatase by OSM and IL-6 in combination with HS1. HS1, 5.0 μg ml$^{-1}$, was prepared from the medium of DX-treated A549 cells and combined with CM, OSM 10 ng ml$^{-1}$ or IL-6 2.5 μg ml$^{-1}$, and AP activity assayed 3 days later. Abbreviations as in previous figures and as in text. Error bars are +s.e.m.

### DISCUSSION

The activity of many growth factors, such as FGF-2 and TGF-β, is regulated by their binding to soluble and membrane-bound low-affinity receptors (Ruoslahti et al, 1992; Lopez-Casillas et al, 1993; de Wynter et al, 1993; Piepkorn et al, 1994; Strain et al, 1994; Walker et al, 1994), many of which are proteoglycans. The role of these receptors is not entirely clear, but they may be involved in sequestration and/or stabilization (McCaffrey et al, 1994), or translocation of the growth factor to the high-affinity receptor (Klagsbrun and Baird, 1991; Ruoslahti et al, 1992). As DX was shown to enhance the activity of growth factors involved in A549 differentiation (although not the activity of FGF-2) (McCormick et al, 1995), and preliminary data suggested that the effect was stable after removal of DX, this suggested that a matrix constituent might be involved, particularly as previous work (McLean, 1986; Mackie et al, 1988) had shown that DX influences the secretion of GAGs by glioma cell lines and by A549.

DX was shown to have a profound effect on secreted and membrane-associated GAGs in A549 cells. Incorporation of [$^3$H]GLN was stimulated in three peaks of activity eluting at 0.1 M, 0.25 M and 0.35 M sodium chloride in subconfluent cells. P1 did not correspond to any of the unlabelled GAGs, and although the increase in P2 corresponded to hyaluronic acid characterization of this peak has not yet been carried out. Although both P1 and P2 were induced by DX in subconfluent cells, they were inhibited by DX in confluent and post-confluent cells. The significance of this reversal is not clear but may be associated with the shift from the high growth fraction associated with subconfluent cultures to the low growth fraction found in confluent cultures.

© Cancer Research Campaign 1997

The 0.35 M sodium chloride peak, designated P3a, corresponded to a low-salt-eluting fraction of heparan sulphate. As previous reports have implicated the heparan sulphate group of proteoglycans in growth factor binding (Klagsbrun and Baird, 1993; de Wynter et al, 1993), this peak was selected for further study. The induction of this peak was seen in both the medium and the trypsinate of subconfluent cells, but not in confluent and post-confluent cultures. Induction in the medium was much greater than that in the trypsinate, in which induction of P3b (0.4 M sodium chloride eluate) was found to be greater. When material from the medium and trypsinate of subconfluent cultures was isolated (designated HS1 from the highest radioactivity fraction in P3a, and HS2 from the middle of P3b) and used instead of DX in the induction of alkaline phosphatase in A549 cells by CM, only HS1 from the medium was found to be active (Figure 3). Cell-associated (trypsinate) HS1 was inactive, and both cell-associated and soluble HS2 were unable to replace DX, although HS2 from the trypsinate and the medium gave 30% and 40% induction with CM, minor effects compared with HS1 from the medium. HS1 from the medium had no inductive activity alone. HS2 from the medium induced AP twofold, in the absence of CM, whether from DX-treated or untreated A549 cells. However, as these effects were slight compared with the effect of HS1 from A549 medium, they were not investigated further.

HS1 was also active in replacing DX in the induction of AP by OSM and IL-6, with HS1 giving a higher response to cytokine than DX in both cases.

Although HS1 isolated from medium and trypsinate was used in the AP assay at the same concentration in control cells, i.e. without DX pretreatment, pooling the d.p.m. per $10^6$ cells from fractions 20–25 showed that about four times as much [$^3$H]GLN incorporation appeared in the medium as in the trypsinate (Table 2). After DX treatment, incorporation in the medium increased 37-fold, whereas that in the trypsinate only increased 2.4-fold, giving a new ratio of medium to trypsinate of 65-fold. As HS1 from the medium was by far the most active, HS1 from the trypsinate, which only increased 2.4-fold with DX as opposed to 37-fold in the medium, was not analysed further.

It is not clear why this activity is not cell associated, as one might expect if it performed the role of a low-affinity translocating receptor, but it may be due to the transformed nature of the A549 cells, where surface proteolysis may release membrane-bound proteoglycans into the medium. Skinner et al (1987) found the ratio of proteoglycans between medium and the cell layer to be similar in rat type II cells in culture, but found that both fractions were reduced with cortisol. Whether this difference is due to species, developmental stage or transformation is not clear.

Prior incubation of HS1 with heparinase or heparitinase, both of which cleave heparan sulphate, destroyed its activity, but incubation with chondroitinase ABC, which is inactive against heparan sulphate but degrades chondroitin sulphate and hyaluronic acid, had no effect. As heparitinase acts specifically on heparan sulphate (Jandik et al, 1994), this suggests that the active material is a species of heparan sulphate. As the purified material was protease treated before fractionation, and only minimal absorbance at 280 nm was observed around the active fractions, suggesting that little residual protein remained, the activity may lie in the carbohydrate moiety. However, the presence and activity of small residual peptide chains cannot be totally excluded.

It is surprising that IL-6 synthesis has been shown to be down-regulated by DX in lung fibroblasts (McCormick et al, 1995), whereas HS1 is stimulated in the target cells that respond to IL-6. However, IL-6 may not be the main paracrine factor from fibroblasts responsible for A549 cell and type II cell differentiation, and the main candidate, FPF, which has been shown to be up-regulated in perinatal lung fibroblasts by cortisol (Post and Smith, 1984), still remains to be identified.

Although many growth factors are dependent on binding to HS for activation, there is no record of this for IL-6 or OSM, although Han et al (1996) observed potentiation of IL-6 by heparin and HS in haematopoietic cells. This may be a new species of HS that interacts with the IL-6 group of cytokines, as we have previously (McCormick and Freshney, 1996) seen DX-dependent activity in LIF as well as OSM and other members of the IL-6 group. However, DX is also required for the inductive action of insulin and IFN-β in this system (McCormick et al, 1995), and it would be surprising to find a common reactive species of HS for such a disparate group. It is possible that each of these agents is capable of inducing the production of an autocrine factor by A549 cells (Stadnyk, 1994). This autocrine factor may be HS dependent and activated by HS1. The current data suggest that untreated A549 cells do not make this autocrine factor constitutively as HS1 is not active alone.

### ABBREVIATIONS

AP, alkaline phosphatase; CM, fibroblast-conditioned medium; DMEM, Dulbecco's modification of Eagle's medium; DX, dexamethasone; F10:DMEM, 50:50 mixture of Ham's F10 and DMEM; FPF, fibrocyte–pneumocyte factor; GAG, glycosaminoglycan; GLN, glucosamine; HA, hyaluronic acid; HS, heparan sulphate; IL-6, interleukin 6; LIF, leukaemia inhibitory factor; OSM, oncostatin M; PNP, *p*-nitrophenol; PS, pulmonary surfactant; SF, serum-free F10:DMEM medium.

### ACKNOWLEDGEMENTS

We are indebted to the Cancer Research Campaign, the Royal Society and the European Association for Cancer Research for support, and to Professor Allan Balmain for useful comments on the manuscript.

### REFERENCES

Bitter T and Muir HM (1962) A modified uronic acid carbazole reaction. *Anal Biochem* **4**: 330–334

Bruce AG, Linsley PS and Rose TM (1992) Oncostatin M. *Prog Growth Factor Res* **4**: 157–170

Caniggia I, Tseu I, Han RN, Smith BT, Tanswell K and Post M (1991) Spatial and temporal differences in fibroblast behavior in fetal rat lung. *Am J Physiol* **261**: 424–433

de Wynter E, Allen T, Coutinho L, Flavell D, Flavell SU and Dexter TM (1993) Localisation of granulocyte macrophage colony-stimulating factor in human long-term bone marrow cultures. *J Cell Sci* **106**: 761–769

Edelson JD, Shannon JH and Mason RJ (1988) Alkaline phosphatase: a marker of alveolar type II cell differentiation. *Am Rev Respir Dis* **138**: 1268–1275

Fernig DG and Gallagher JT (1994) Fibroblast growth factors and their receptors: an information network controlling tissue growth. Morphogenesis and repair. *Prog Growth Factor Res* **5**: 353–377

Giard DJ, Aaronson SA, Todaro GJ, Arnstein P, Kersey JH, Dosik H and Parks WO (1972) In vitro cultivation of human tumours: establishment of cell lines from a series of solid tumours. *J Natl Cancer Inst* **51**: 1417

Han ZC, Bellucci S, Shen ZX, Maffrand JP, Pascal M, Petitou M, Lormeau J and Caen JP (1996) Glycosaminoglycans enhance megakaryocytopoiesis by modifiying the activities of hematopoietic growth regulators. *J Cell Physiol* **168**: 97–104

© Cancer Research Campaign 1997

Hawgood S and Clements JA (1990) Pulmonary surfactant and its apoproteins. *J Clin Invest* **86**: 1–6

Jandik KA, Gu KA and Linhardt RJ (1994). Action pattern of polysaccharide lyases on glycosaminoglycans. *Glycobiology* **4**: 289–296

Juricova M and Deyl Z (1975) Polysaccharide-protein complexes. In: *Liquid Column Chromatography. A Survey of Modern Techniques and Applications*, (Deyl Z, Macek K and Janak J), pp. 529–542. Elsevier: Amsterdam,

Klagsbrun M and Baird A (1991) A dual receptor system is required for basic fibroblast growth factor activity. *Cell* **67**: 229–231

Kohlhepp EA, Condon ME and Hamburger AW (1987) Recombinant human interferon alpha enhancement of retinoic acid induced differentiation of HL-60 cells. *Exp Hematol* **15**: 414–418

Lieber M, Smith B, Szakal A, Nelson Rees W and Todaro G (1976) A continuous tumour-cell line from a human lung carcinoma with properties of type II alveolar epithelial cells. *Int J Cancer* **17**: 62–70

Lopez-Casillas F, Wrana JL and Massague J (1993) Betaglycan presents ligand to the TGF-β signalling receptor. *Cell* **73**: 1435–1444

Lotem J and Sachs L (1995) Regulation of blc-2, bcl-x and bax in the control of apoptosis by hematopoietic cytokines and dexamethasone. *Cell Growth Diff* **6**: 647–653

McCaffrey TA, Falcone DJ, Vicente D, Du B-h, Consigli S and Borth W (1994) Protection of transforming growth factor-β1 activity by heparin and fucoidan. *J Cell Physiol* **159**: 51–59

McCormick C, Freshney RI and Speirs V (1995) Activity of interferon-α, interleukin-6 and insulin in the regulation of differentiation in A549 alveolar carcinoma cells. *Br J Cancer* **71**: 232

McCormick C and Freshney RI (1996) Activity of growth factors in the IL-6 group for the differentiation of A549 human lung adenocarcinoma. Ms in preparation

Mackie AE, Freshney RI, Akturk F and Hunt G (1988) Glucocorticoids and the cell surface of human glioma cells: Relationship to cytostasis. *Br J Cancer* **58**(suppl. IX): 101–107

McLean J (1986) *The Modulation of the Phenotype of Human Non-Small-Cell Lung Carcinoma*. PhD Thesis, University of Glasgow

Oberg K (1992) The action of interferon alpha on human carcinoid tumours. *Cancer Biol* **3**: 35–41

Pfeffer LM and Eisenkfraft BL (1991) The antiproliferative and antitumour effects of human alpha interferon on cultured renal carcinomas correlate with the expression of a kidney-associated differentiation antigen. *Interferons Cytokines* **17**: 30–31

Piepkorn M, Lo C, Plowman G (1994). Amphiregulin dependent proliferation of cultured human keratinocytes: autocrine growth, the effects of exogenous recombinant cytokine and apparent requirement for heparin like glycosaminoglycans. *J Cell Physiol* **159**: 114–120

Post M and Smith BT (1984) Effect of fibroblast-pneumocyte factor on the synthesis of surfactant lipids in type II cells from fetal rat lung. *Biochim Biophys Acta* **793**: 297–299

Post M, Floros J and Smith BT (1984) Inhibition of lung maturation by monoclonal antibodies against fibroblast-pneumocyte factor. *Nature* **308**: 284–286

Rose TM and Bruce AG (1991) Oncostatin M is a member of a cytokine family that includes leukaemia-inhibitory factor, granulocyte colony-stimulating factor and interleukin 6. *Proc Natl Acad Sci USA* **88**: 8645–8645

Ruoslahti E, Yamaguchi Y, Hilderbrand A and Border WA (1992) *Extracellular Matrix/Growth Factor Interactions* Vol. LVII, pp. 309–314. Cold Spring Harbor Sym. Quant. Biol: Cold Spring Harbor, NY

Skinner SJM, Post M, Torday JS, Stiles AD and Smith BT (1987) Characterization of proteoglycans synthesized by fetal-rat lung type-II pneumonocytes in vitro and the effects of cortisol. *Exp Lung Res* **12**: 253–264

Speirs V, Ray KP and Freshney RI (1991) Paracrine control of differentiation in the alveolar carcinoma, A549, by human foetal lung fibroblasts. *Br J Cancer* **64**: 693–699

Stadnyk AW (1994) Cytokine production by epithelial cells. *FASEB J* **8**: 1041–1047

Strain AJ, McGuiness G, Rubin JS and Aaronson SA (1994) Keratinocyte growth factor and fibroblast growth factor action on DNA synthesis in rat and human hepatocytes: modulation by heparin. *Exp Cell Res* **210**: 253–259

Walker A, Turnbull JE and Gallagher JT (1994) Specific heparan sulphate saccharides mediate the activity of basic fibroblast growth factor. *J Biol Chem* **269**: 931–935

© Cancer Research Campaign 1997